# Innovative Use of Nanomaterials in Treating Retinopathy of Prematurity

**DOI:** 10.3390/ph17101377

**Published:** 2024-10-16

**Authors:** Kevin Y. Wu, Xingao C. Wang, Maude Anderson, Simon D. Tran

**Affiliations:** 1 Department of Surgery, Division of Ophthalmology, University of Sherbrooke, Sherbrooke, QC J1G 2E8, Canada; yang.wu@usherbrooke.ca (K.Y.W.);; 2 Faculty of Medicine and Health Sciences, McGill University, Montreal, QC H3T 1J4, Canada; 3 Faculty of Dental Medicine and Oral Health Sciences, McGill University, Montreal, QC H3A 1G1, Canada

**Keywords:** biomaterials, nanomaterials, drug delivery, slow release, biocompatibility, retinopathy of prematurity, drug delivery systems (DDSs), nanosystems, nanoparticles, nanotechnology, nanomedicine

## Abstract

Background/Objectives: Retinopathy of prematurity (ROP) is a severe condition primarily affecting premature infants with a gestational age (GA) of 30 weeks or less and a birth weight (BW) of 1500 g or less. The objective of this review is to examine the risk factors, pathogenesis, and current treatments for ROP, such as cryotherapy, laser photocoagulation, and anti-VEGF therapy, while exploring the limitations of these approaches. Additionally, this review evaluates emerging nanotherapeutic strategies to address these challenges, aiming to improve ROP management. Methods: A comprehensive literature review was conducted to gather data on the pathogenesis, traditional treatment methods, and novel nanotherapeutic approaches for ROP. This included assessing the efficacy and safety profiles of cryotherapy, laser treatment, anti-VEGF therapy, and nanotherapies currently under investigation. Results: Traditional treatments, while effective in reducing disease progression, exhibit limitations, including long-term complications, tissue damage, and systemic side effects. Nanotherapeutic approaches, on the other hand, have shown potential in offering targeted drug delivery with reduced systemic toxicity, improved ocular drug penetration, and sustained release, which could decrease the frequency of treatments and enhance therapeutic outcomes. Conclusions: Nanotherapies represent a promising advancement in ROP treatment, offering safer and more effective management strategies. These innovations could address the limitations of traditional therapies, reducing complications and improving outcomes for premature infants affected by ROP. Further research is needed to confirm their efficacy and safety in clinical practice.

## 1. Introduction

Retinopathy of prematurity (ROP) is a critical condition primarily affecting premature infants with a gestational age (GA) of 30 weeks or less and a birth weight (BW) of 1500 g or less. This review provides an exploration of ROP, beginning with an overview of the risk factors, pathogenesis, and retinal vascular development. Central to understanding ROP is the interplay between the vascular endothelial growth factor (VEGF) and insulin-like growth factor I (IGF-I) in terms of abnormal retinal blood vessel growth, due to the hyperoxic environments and hypoxia-induced neovascularization experienced by premature infants [1].

Traditional and current management strategies for ROP include cryotherapy, laser photocoagulation, and anti-VEGF therapy [2]. Cryotherapy, though effective in reducing unfavorable outcomes, is associated with significant long-term complications. Laser photocoagulation, which ablates the peripheral avascular retina, is preferred over cryotherapy, but still presents challenges, including potential damage to surrounding tissues, as well as ocular complications. Anti-VEGF therapy has shown promise in treating ROP, but raises concerns regarding systemic side effects and long-term safety, as these agents can circulate beyond the eye and impact other developing organs [3,4,5].

The limitations of these current treatments have spurred interest in exploring new methods, particularly nanotherapeutic approaches. Nanotherapies offer several potential advantages, including targeted delivery to reduce systemic and ocular toxicity, enhanced permeability and retention for better drug delivery to retinal tissues, and controlled, sustained release of medications [6]. These properties can potentially reduce the frequency of injections and increase therapeutic effectiveness, promising a more effective and safer alternative for managing ROP. This review delves into these nanotherapeutic approaches, examining how innovations in nanosystems could potentially revolutionize the treatment landscape for ROP.

## 2. Overview of Retinopathy of Prematurity (ROP)

Retinopathy of prematurity (ROP) is primarily associated with two major risk factors: a gestational age (GA) of 30 weeks or less and a birth weight (BW) of 1500 g or less. Premature infants born with a significantly lower GA are at a higher risk of developing ROP, due to the underdevelopment of their retinal vasculature. Additionally, infants with a low BW are more predisposed to ROP, as their underdeveloped systems are more susceptible to complications affecting retinal growth. Excessive supplemental oxygen during the early postnatal period is a notable risk factor for ROP, as it disrupts normal retinal vascularization. Additionally, low serum IGF-I levels correlate with poor postnatal weight gain and more severe manifestations of ROP [7].

Retinal vascular development begins around 16 weeks of gestation, with mesenchymal tissue, the source of retinal vessels, growing outward from the optic disc and reaching the nasal ora serrata by 36 weeks and the temporal ora serrata by 40 weeks. In premature infants, ROP occurs due to abnormal retinal blood vessel growth, driven by the interplay between vascular endothelial growth factor (VEGF) and insulin-like growth factor I (IGF-I). The pathogenesis of ROP starts with the hyperoxic environment (i.e., supplemental oxygen) often required for these infants, disrupting normal blood vessel growth and subsequently leading to ischemia. Specifically, during the first phase, between 22 and 30 weeks of gestational age, the retina experiences relative hyperoxia compared to intrauterine oxygen levels, resulting in low VEGF levels and the cessation of retinal blood vessel growth, exacerbated by high oxygen levels, reduced IGF-I levels, and poor weight gain. In the second phase, between 31 and 44 weeks, the avascular retina becomes hypoxic, triggering the release of VEGF. This increase in hypoxia-induced VEGF leads to abnormal neovascularization on the retinal surface, causing complications, such as retinal detachment, which is a critical aspect of ROP progression [1].

ROP is classified based on the location, stage, extent of the disease, presence of plus disease, and the aggressiveness of the condition. The zone classification defines the location, with Zone I being the area around the optic nerve extending to twice the distance from the macula to the optic nerve, Zone II extending to the nasal ora serrata, and Zone III encompassing the remaining temporal peripheral retina [8].

**Figure 1:**
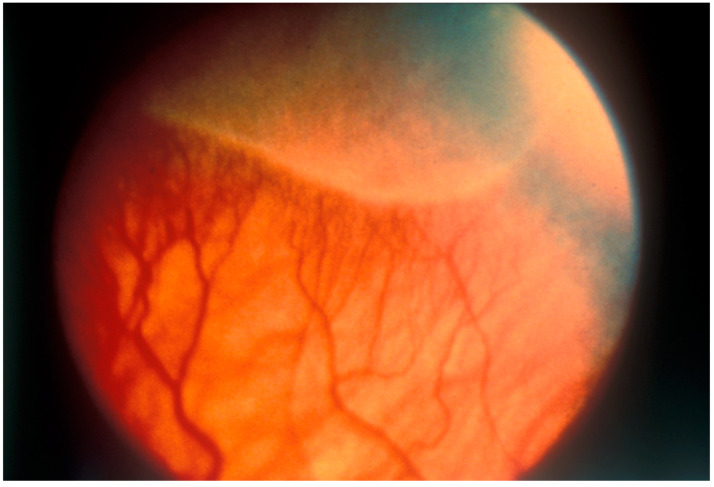
This fundus photograph illustrates Stage 1 disease, where the boundary between the central, vascularized retina, and the peripheral, avascular retina is clearly visible. Gilbert C et al. (1998, updated 2007). Prevention of childhood blindness teaching set. London: International Centre for Eye Health www.iceh.org.uk, (accessed on 5 May 2024) licensed under Creative Commons CC BY-NC 2.0.

**Figure 2:**
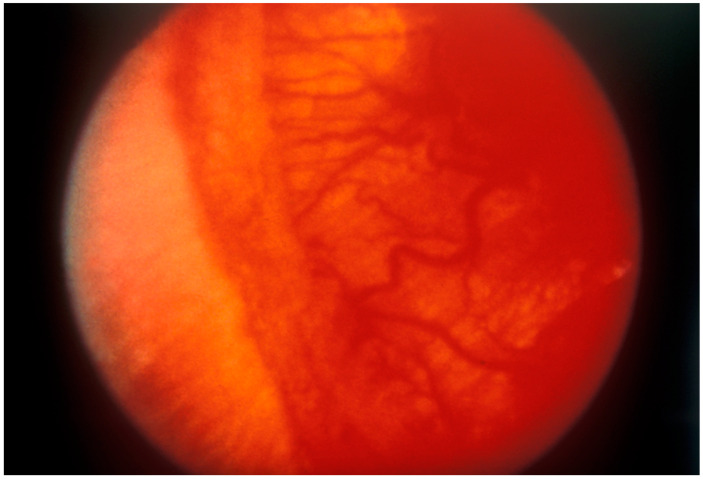
Stage 3 ‘plus’ disease is depicted with extensive fibrovascular proliferation and prominently dilated, tortuous retinal blood vessels. Gilbert C et al. (1998, updated 2007). Prevention of childhood blindness teaching set. London: International Centre for Eye Health www.iceh.org.uk, (accessed on 5 May 2024) licensed under Creative Commons CC BY-NC 2.0.

The stage classification indicates severity: Stage 1 is characterized by a demarcation line between the vascularized and avascular retina (Figure 1); Stage 2 involves a ridge with popcorns or tufts; Stage 3 includes fibrovascular proliferations extending into the vitreous (Figure 2); Stage 4 is partial retinal detachment, further categorized into 4A (extrafoveal retinal detachment) and 4B (involvement of the fovea); and Stage 5 denotes complete retinal detachment [8].

The presence of plus disease is identified by venous dilation and arteriolar tortuosity in the posterior retinal vessels (Figure 2), along with additional signs, such as iris vascular dilatation (rubeosis) and vitreous haze. The extent of the disease is measured by the number of clock hours affected, indicating the circumferential extent of the disease. Aggressive ROP (AROP), also known as Rush Disease, is characterized by the rapid progression of plus disease located in Zone I or the very posterior Zone II, necessitating urgent intervention due to its swift advancement [8].

The prognosis for infants with ROP is generally positive, with most cases (approximately 90%) resolving fully without the need for treatment. A sign of regression includes a clear zone of retina beyond the shunt, followed by the development of straight vessels crossing the shunt, and an arteriovenous feeder extending into the avascular retina. This indicates that the abnormal blood vessel growth has ceased and the retina is healing [2].

However, ROP can lead to various complications, particularly from late sequelae of regressed ROP. These complications include ocular conditions, such as myopia with astigmatism, anisometropia, strabismus, and amblyopia. Structural issues can also arise, including vitreoretinal scarring, an abnormal vitreoretinal interface and adhesions, recurrent vitreous hemorrhages, and different types of retinal detachments (tractional, rhegmatogenous, and exudative). Additionally, anomalous foveal anatomy and macular pigment epitheliopathy can occur several years after the treatment and resolution of ROP [9,10].

Other potential complications include the development of cataracts and glaucoma. These conditions require ongoing monitoring and management to ensure that the child’s vision and eye health are preserved, as much as possible. Early detection and treatment of these complications are crucial for minimizing long-term visual impairment and improving the overall quality of life of the affected individuals [9,10].

Given the potential severity of ROP and its complications, screening is a critical component of managing this condition. Infants with a birth weight of less than 1500 g (or less than 1250 g in Canada) and those with a gestational age of 30 weeks or less should be screened for ROP. Additionally, screening should be performed based on the neonatologist’s recommendation for any birth weight or gestational age [11,12].

The screening protocol includes specific guidelines: screening should never be conducted before 31 weeks of postmenstrual age, due to the fact that corneal haze can obstruct the view of the fundus, and it should be performed 4 weeks after birth, since ROP typically develops at least 4 weeks post-birth in the context of supplemental oxygen therapy. This structured approach to screening helps ensure early identification and timely intervention, which are essential for preventing the progression of ROP and reducing the risk of visual impairment [11,12].

## 3. Managing Retinopathy of Prematurity

Approximately 10% of infants screened for retinopathy of prematurity (ROP) require treatment. The decision to treat is based on the location and severity of the disease, classified into type 1 and type 2 ROP [4].

Type 1 ROP, which usually requires treatment, is defined by the following criteria: in Zone I, either Stage 1 or 2 ROP with plus disease, or Stage 3 ROP without plus disease; and in Zone II, Stage 2 or 3 ROP with plus disease, irrespective of the extent of the disease. The current guidelines strongly recommend treatment for any eye with type 1 ROP [4].

In contrast, type 2 ROP does not necessitate immediate treatment, but requires close monitoring. Type 2 ROP is defined by the presence of Stage 1 or 2 ROP without plus disease in Zone I, or Stage 3 ROP without plus disease in Zone II. Eyes with type 2 ROP should be closely observed for progression to type 1 disease to ensure timely intervention if necessary [4].

### 3.1. Cryotherapy

Several multicenter trials have significantly influenced the treatment protocols for ROP. The initial study, CRYO-ROP (Multicenter Trial of Cryotherapy for Retinopathy of Prematurity), recommended using cryotherapy when the disease reached a defined level of severity, known as the threshold disease [3]. Although cryotherapy and the concept of threshold disease are no longer used clinically, with current standards shifting towards intravitreal anti-VEGF injections and laser photocoagulation, the CRYO-ROP study laid the groundwork for subsequent research and advancements in the treatment of ROP.

Cryotherapy hinders abnormal growth by applying extremely low temperatures to the retina. This treatment modality was first deemed effective following the CRYO-ROP study, which demonstrated a reduction in unfavorable outcomes by half over three months [13]. The 10-year follow-up revealed that the incidence of total retinal detachment continued to increase in the control eyes from 5 ½ years post-treatment and onwards, whereas it remained stable in cryotherapy-treated eyes. However, the treated eyes were still highly associated with poor outcomes relating to visual acuity, with a 10-year follow-up indicating that there was a similar percentage of treated and control eyes with 20/40 or better visual acuity. The 15-year outcomes report highlighted a gradual increase in unfavorable structural outcomes as time progressed; 4.5% of treated and 7.7% of control eyes developed new unfavorable results, such as retinal detachment. This result underscores the need for long-term follow-up post-cryotherapy treatment. Further research focusing on myopia as an outcome of cryotherapy demonstrated that both laser and cryotherapy treatments caused myopia, though it worsened up to 3 years after treatment using cryotherapy [14]. Cryotherapy also has significantly more retinal dragging than its comparison, which could explain the poorer visual acuity, juxta macular chorioretinal scars, loss of cilia, and blepharoptosis that were also outcomes observed in treated patients [15].

### 3.2. Laser Photocoagulation

The Early Treatment for Retinopathy of Prematurity (ETROP) trial demonstrated that initiating treatment earlier in pre-threshold eyes classified as type 1 leads to improved structural and visual outcomes compared to conventional treatment [4]. Panretinal laser photocoagulation, which ablates the peripheral avascular retina, is the recommended treatment. This method is favored over cryotherapy due to the inconveniences and negative outcomes associated with the latter, as discussed in the previous section. The current guidelines strongly advocate for treatment of any eye diagnosed with type 1 ROP [4].

Laser treatment halts the growth of abnormal blood vessels in the retina through laser burns. More precisely, it is a form of ablation therapy used to prevent an increase in VEGF by destroying the avascular retina. As portable indirect laser photocoagulation machines become more accessible, this treatment modality has shown great potential and is the preference when compared to cryotherapy [16]. The benefits of laser treatment include the ability to use topical anesthetics compared to general anesthesia, which could eliminate the risks and potential complications of general anesthesia [17]. Furthermore, Ng et al. demonstrated that eyes treated with lasers had more optimal best-corrected visual acuity and less retinal dragging than cryotherapy [15]. Conversely, when looking at more aggressive posterior ROP, though disease refreshment was achieved, laser treatment resulted in poor visual outcomes. Moreover, cataracts most likely caused by secondary anterior segment ischemia are complications that can have a high impact on visual acuity [18,19,20]. Recently, Ajjarapu et al. (2023) have suggested the possible occurrence of late-onset anterior segment complications following laser therapy, expressing clinical symptoms such as band keratopathy, cataracts, glaucoma, pupillary membrane, and posterior synechia, causing some to undergo additional procedures to resolve these complications. Many patients have also experienced progressive myopia, which alongside band keratopathy or corneal irregularities, prevents them from wearing contact lenses. These adverse effects can result in detrimental effects to visual acuity; hence, the importance of long-term follow-up due to these complications arising an average of 8.7 years post-treatment [21].

### 3.3. Anti-Vascular Endothelial Growth Factor Therapy

The latest treatment for type 1 retinopathy of prematurity (ROP) involves intravitreal injections of anti-VEGF agents, specifically bevacizumab and ranibizumab. The landmark study in this area, “Bevacizumab Eliminates the Angiogenic Threat of Retinopathy of Prematurity” (BEAT-ROP), demonstrated a significant improvement in the structural outcomes for zone I eyes treated with intravitreal bevacizumab monotherapy compared to laser treatment. Anti-VEGF therapy consists of injections of drugs inhibiting the vascular endothelial growth factor (VEGF), which promotes vascular damage and the growth of abnormal blood vessels in hypoxic environments [22]. Unlike laser photocoagulation, which is often more time consuming, requires specific equipment, and trained ophthalmologists, anti-VEGF therapy it is more attractive for developing countries [23]. Furthermore, there is evidence that this method addresses the myopic outcome and minimizes peripheral visual field loss [5]. Nonetheless, retinal detachment, seen in cryotherapy, is also seen in this method. Peripheral and posterior lesions, like a massive loss of the retinal capillary bed, otherwise less appreciated in laser therapy, highlight the uncertainty in terms of the long-term effects of anti-VEGF therapy [24]. This might explain why recent studies have shown that retinopathy of prematurity (ROP) can recur several months post-treatment with anti-VEGF agents, necessitating extended monitoring and possible re-treatment following intravitreal injections [5]. Consequently, anti-VEGF therapy is not recommended for infants unlikely to return for regular follow-up visits after hospital discharge. Jalali et al. (2013) performed a retrospective study and reported ocular adverse effects, including retinal breaks, vascular attenuation, and retinal pigment epithelial (RPE)/choroidal rupture [23].

Concerns have been raised that the impact of antiangiogenic drugs on the developing vasculature elsewhere in the body could result in adverse developmental outcomes. Multiple studies have demonstrated that intravitreal injections of anti-VEGF, specifically bevacizumab, decrease serum VEGF levels for around 2 months, indicating potential systemic circulation of bevacizumab post-injection. In vivo experiments on rat pups have shown supporting evidence of systemic circulation, such as weight loss [25]. VEGF signalling in the lungs and kidneys was also abnormal, consequently affecting the pulmonary vasculature and causing pulmonary hypertension, right ventricular hypertrophy, and increased heart weight [26]. Moreover, hepatic dysfunction, indicated by liver enzyme levels five times higher than normal, highlights the potential systemic toxicity of this therapy [23]. Therefore, it is crucial to acknowledge the importance of VEGF in the development of various organs and how potential systemic toxicity from anti-VEGF therapy may impact vital organs [27,28]. On the topic of the neurological system, anti-VEGF therapy, specifically bevacizumab, has shown mixed effects on neurodevelopmental outcomes in human studies. While some studies suggest lower cognitive and motor skills in children treated with bevacizumab, others have concluded that these differences are insignificant when compared to laser treatment [29,30]. In summary, the long-term ocular and systemic effects of anti-VEGF agents in the treatment of ROP require further investigation.

### 3.4. Current Challenges in Managing Retinopathy of Prematurity

The challenges associated with cryotherapy, laser photocoagulation, and anti-VEGF injections have prompted researchers to explore new treatment methods for ROP. Nanotherapeutic approaches offer potential advantages, including targeted delivery to reduce systemic and ocular toxicity, and enhanced permeability and retention for better drug delivery to retinal tissues. Additionally, these nanotherapies provide controlled and sustained release of medications, potentially reducing the frequency of injections and increasing therapeutic effectiveness. These innovations potentially offer a more effective and safer alternative for managing ROP. The next section will dive indepth into these nanotherapeutic approaches.

## 4. Nano-Based Therapeutic Approaches

### 4.1. Various Nano-Based Drug Delivery Systems

**Figure 3:**
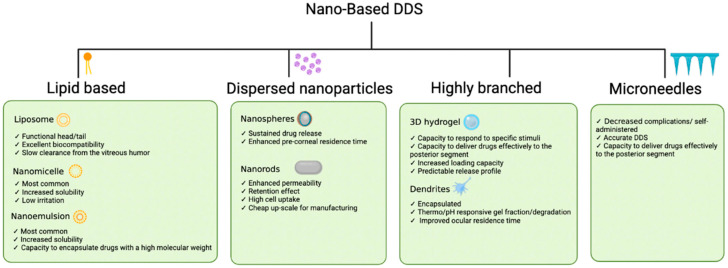
A summary of the characteristics and properties of important nano-based drug delivery systems used in ocular drug delivery.

There are various forms of nanotechnology-based drug delivery systems (DDSs) used to achieve long-lasting and specific delivery of drugs to select parts of the eye. Their characteristics and advantages differ based on their materials and composition, which are discussed in Figure 3.

### 4.2. Lipid Nanoparticles

**Figure 4:**
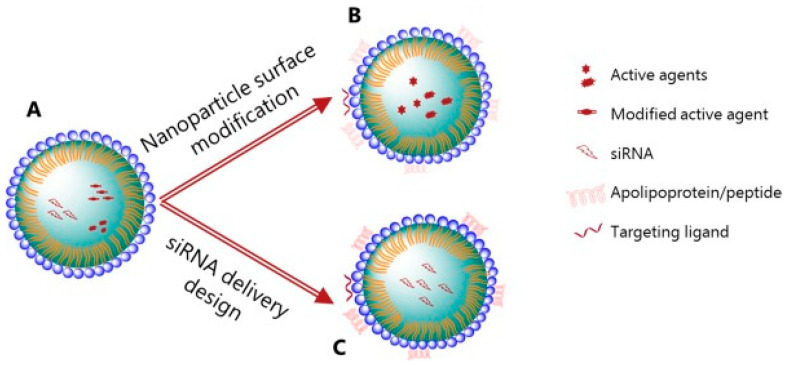
Lipid nanoparticles in delivery of therapeutics. (**A**) Plain lipid NP, where the core can be of liquid, solid crystalline, or lyotropic liquid form. (**B**) Lipid NP with surface modifications for enhanced delivery of therapeutics through receptor-mediated targeting. (**C**) Lipid NPs containing siRNA with targeting properties for efficient delivery of therapeutics. Disclaimer: Reprinted with permission from ScienceDirect, Copyright 2024, under a Creative Common License, CC BY 4.0, https://creativecommons.org/licenses/by/4.0/ (accessed on 5 May 2024), Advanced Drug Delivery Reviews, Vol 203, Yaghmur et al., “Lipid nanoparticles for targeted delivery of anticancer therapeutics: Recent advances in development of siRNA and lipoprotein-mimicking nanocarriers”, no changes made [37].

Lipid-based nanoparticles are widely known for their versatility, transportation of both hydrophobic and hydrophilic molecules, stability, and low-cost production [31,32]. In the context of retinopathy of prematurity, Bohley et al. (2022) employed the targeting advantage of lipid NPs to address the issue of systemic toxicity in intravenous drug delivery [33]. Though intravenous injections are more accessible compared to current gold standard treatments, their potential is limited by the multiple barriers that would need to be crossed before reaching the retinal epithelial layer. Permeability to choroid capillaries and the Burch membrane is essential. To address this limitation, lipid nanocapsules (LNCs) were used due to their resemblance to VLDLs and their inherent ability to cross the necessary barriers. To minimize systemic toxicity, the LNPs were coated with cyclo(-Arg-Gly-Asp-D-Phe-Cys) (cRGD), a ligand targeting RPE cells. Fluorescence microscopy and transmission electron microscopy (TEM) analysis proved the LNCs’ ability to pass through barriers, following the same path as VLDL molecules, but that only RGD-LNCs accumulated sufficiently in the target tissue, demonstrating the targeting properties of cRGD in RPE cells. The incorporation of cyclosporin A (CsA), for its antiangiogenic and anti-inflammatory effects, produced encouraging results, following a single injection of CsA RGD-LNCs in vivo in an oxygen-induced retinopathy mouse model [34]. Wang et al. (2020) took this a step further by combining lipid-like NPs and gene therapy, addressing the issue of nuclease degradation and unwanted immune activation following exposure to naked RNA therapy. The lipidoid materials were intentionally composed of disulphide bonds, which have the unique property of degrading when exposed to GSH-rich environments that are often found intracellularly [35]. By incorporating VEGF siRNA in lipid NPs, a successful reduction in hypoxia-induced VEGF expression was seen in oxygen-induced retinopathy (OIR) rat models. Its effect on retinal neovascularization is also comparable to that of ranibizumab therapy, a widely recognized current treatment modality. Despite positive outcomes, there remains a need for long-term follow-up to identify possible important complications that may arise [36]. The incorporation of smart NPs allows for the intracellular release of siRNA, but no analysis has been conducted on the targeting properties of such NPs and possible systemic outcomes. Figure 4 illustrates the various lipid nanoparticle modifications discussed in this section, including ligand-targeting and gene therapy.

### 4.3. Gold Nanoparticles

It is known that gold nanoparticles have angiogenic properties in vivo and are capable of crossing the blood–retinal barrier, hence making it a candidate for the intravenous treatment of neovascularization pathologies, such as ROP [38,39]. Kim et al. (2011) presented the inhibitory properties of independent gold nanoparticles in terms of retinal neovascularization. GNPs inhibited the proliferation, migration, and tube formation in retinal microvascular endothelial cells when studied in vitro, and the intravitreal injection of GNPs in vivo inhibited retinal neovascularization in OIR mice via the suppression of the VEGFR-2 signalling pathway. Additionally, GNPs did not affect cellular viability up to 7 days following injection, indicating a low possibility of toxicity to retinal cells. Furthermore, histological examination of the retinal layers showed no significant thickness changes and the absence of inflammatory cells [40]. Song et al. (2017) also used the self-therapeutic properties of gold NPs to their advantage, producing gold nanodiscs with diagnostic and therapeutic properties. Due to the nanodiscs’ anatomical structure, they can be picked up on OCT scans at a 100 times lower concentration than rod-shaped GNPs. Additionally, these discs displayed anti-angiogenic and biocompatible properties in vivo. However, the potency and specificity of gold nanodiscs are obstacles, as their attraction to VEGF relies solely on electrostatic interactions [41]. The potential of gold nanoparticles is immense, with possibilities in terms of dual functions. However, Söderstjerna et al. (2014) analyzed the possible adverse effects of gold and silver nanoparticles in retinal cells and found that both types of NPs caused morphological and physiological changes to the retina and its constituents. For example, NPs lead to an increase in retinal cell apoptosis, especially in the photoreceptor layer and the presence of vacuoles in the inner retinal layer. GFAP upregulation, a hallmark of retinal injury, was also observed following exposure to NPs [42]. The size, shape, and surface coating of gold nanoparticles all play a role in its toxicity. Hence, the extent of our understanding when it comes to GNP-optimized compositions and its effects on overall health remains uncertain and requires further investigation, before considering its clinical potential in children.

### 4.4. Polymeric Nanoparticles

Polymeric nanoparticles can be made from various building materials, such as poly(lactide-co-glycolide) (PLGA), poly(lactic acid) (PLA), and poly(ethylene glycol) (PEG), amongst others. The advantages of such NPs includes their increased bioavailability, the related therapeutic index, and their ability to protect drugs/molecules from the environment [43]. It has been identified that the in vitro delivery of pazopanib through PLGA and PLA blocked retinal microvascular endothelial cell migration and proliferation, thus inhibiting their angiogenic potential [44]. Zhang et al. (2018) encapsulated bevacizumab in PLGA NPs in the hope of increasing its bioavailability, mitigating the need for frequent intravitreal injections due to the short half-life of the free drug. PLGA NPs have demonstrated long-lasting and controlled release of bevacizumab, where 40% of the drug was released in 7 days, whereas almost all free bevacizumab was released in the first 2–7 h. Moreover, bevacizumab-encapsulated PLGA NPs failed to display toxicity in regard to the retina or choroid and provided better inhibition of angiogenesis in OIR mice, as seen by the smaller areas of neovessel formation compared to free-drug injections [45]. Moving away from ROP phase 2 treatments, Mezu-Ndubuisi et al. (2019) explored the potential of VEGF-A165-encapsulated PLGA NPs in phase 1 ROP. Phase 1 is characterized by the delay in vascular growth, whereas phase 2 is the abnormal vaso-proliferation phase [1]. VEGF-A165 is a variant of VEGF-A, with pro-angiogenic properties, and its sustained release in phase I ROP was shown to promote vascular regeneration in OIR mice, while reducing venous dilation and arterial tortuosity and preventing severe ROP [46,47].

## 5. Clinical Barriers and Future Perspectives

### 5.1. Commercial Interest

Retinopathy of prematurity is currently a leading cause of avoidable childhood blindness in low-and middle-income countries. High-income countries have access to optimized screening programs and access to adequate facilities, equipment, and staff to provide the appropriate treatment currently in place [48]. Hence, there is a lack of commercial interest in developing new ROP treatment modalities [34]. In return, this limits the numerous advantages of NPs that could benefit all patients, such as increased bioavailability and the elimination of systemic toxicity. Furthermore, current investigations on nanoparticles in ROP are limited and have yet to advance past the preclinical stage. Although retinopathy of prematurity shares similar pathophysiology as other ischemic neovascular diseases, such as diabetic retinopathy, the results from studies looking at the wider theme of ischemic neovascular diseases cannot be directly translated to pediatrics [7]. Further, inside the realm of pediatrics, various age groups differ in terms of their physiology and metabolism [49]. Therefore, there remains a strong need to explore the topic of ROP, whilst considering the unique physiology of infants. Though research relating to children is more challenging to conduct, due to funding, the uniqueness of children, and particular ethical concerns, among others, these studies remain crucial in better understanding the efficacy and potential harms of such novel treatments [50].

### 5.2. Retinal Pigment Epithelium Challenges and Potential

**Figure 5:**
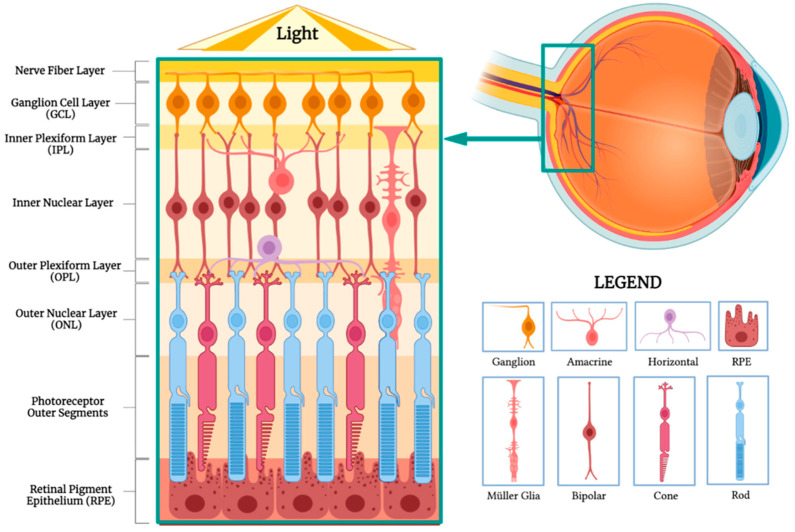
The structural layers of the retina and their respective contents.

The retinal pigment epithelium plays a crucial role in maintaining retinal homeostasis, while producing mediators of inflammation, immune system activation, and neovascularization. As mentioned previously, intravitreal and intravenous injections are the most common drug delivery systems currently studied. However, such modalities require the NPs to cross various barriers (Figure 5). Following an intravitreal injection, the NPs first encounter the nerve fiber layer composed of ganglion cell axons, which form the optic nerve, followed by the ganglion cell layer containing their cell bodies. These specialized cells then synapse with bipolar cells in the inner plexiform layer, which then transmit the visual information to the cell bodies of amacrine, bipolar, and horizontal cells. The outer plexiform layer is where these cells synapse with cones and rods. The bodies of photoreceptors are found in the outer nuclear layer and extend to the photoreceptor outer segments, after which the RPE is finally reached. During the intravenous route, NPs encounter the choroid and Burch’s membrane, before arriving at the RPE [51]. Up until now, specific RPE-targeting therapies for ROP remain mostly unexplored. The majority of studies focus on reducing VEGF. Although Bohley et al. (2022) demonstrated successful RPE targeting, there remains a general lack of investigations on RPE-specific drug delivery and diffusion systems [34]. Further, the ideal design in terms of particle size, charge, and composition, remains inconclusive. Lastly, it is important to consider the differences between rodent and human anatomy. As current research on NPs in ROP is mainly preclinical in vivo experiments on mice, it is crucial to acknowledge that the current results are not guaranteed to be directly applicable to humans, more specifically children. For example, rodent inner limiting membranes are thinner and less complex than humans, which could optimize NP delivery results that may not translate clinically [52].

## 6. Emerging Trends

### 6.1. Gene Therapy

Nanoparticles hold the potential to deliver gene therapy in retinopathy of prematurity. Antisense oligonucleotides (ODNs) can alter RNA, thus modifying protein expression [53]. Hagigit et al. (2012) found that anti-VEGFR oligonucleotides in DOTAP cationic nanoemulsions enhanced the efficacy of the ODN and that this may be due to increased intracellular uptake or stability of the ODN due to protection by the nanocarriers [54]. Using folic acid–chitosan-modified mesoporous silica nanoparticles, Huang et al. (2022) loaded them with MiRNA-223, which induced the phenotypic transition of microglial to the anti-inflammatory state (M2), increasing anti-inflammatory cytokines, which caused a decrease in over 50% of the retinal neovascular area in vivo. The folic acid targeted M1 microglia, whereas chitosan enhanced cell membrane penetration due to its positive charge. Histological analysis did not indicate any signs of toxicity in terms of the retina or major organs, including the liver, spleen, or kidney [55]. Finally, NPs can also be a means of transportation for plasmids, allowing the incorporation of novel elements into the host. Non-specific to ROP, the encapsulation of plasmids in PLGA NPs to moderate neovascularization has been investigated to inhibit angiogenesis [56]. Wang et al. (2015) incorporated the plasmid very low-density lipoprotein receptor extracellular domain (VLN) in PLGA NPs, following a hypothesis that VLN inhibits WNT signalling and VEGF as its downstream result [57]. In short, nanoparticles show great potential in addressing obstacles in gene therapy, which have hindered their full potential in the realm of retinopathy of prematurity. By addressing these obstacles, nanoparticles present the opportunity for gene therapy to be used as a novel treatment in ROP.

### 6.2. Exosomes

**Figure 6:**
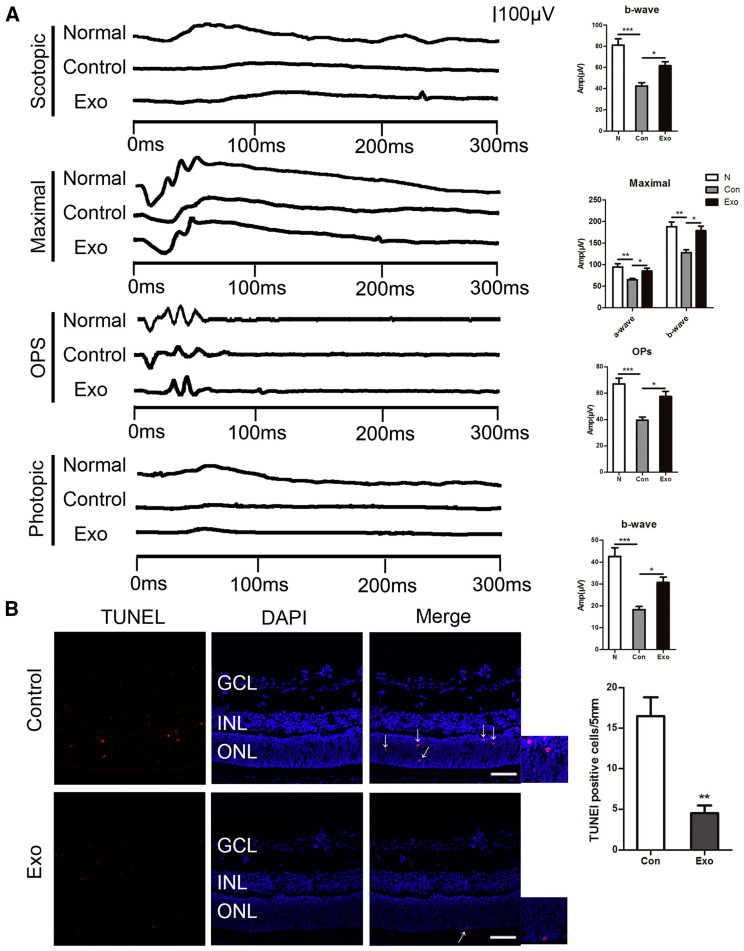
(**A**,**B**) Microglia-derived exosomes benefit visual function, whilst reducing retinal photoreceptor apoptosis in OIR mice. (**A**) Measurements of scotopic electroretinography (ERG), maximal response, OP, and photopic ERG in injected group, control group, and normal group. The b-wave amplitude of scotopic and photopic ERG, a-, and b-wave of maximal response, and OP P3 amplitude were compared amongst the 3 groups. (**B**) Images of retinal cryosections with TUNEL (Red) staining of microglia-derived exosomes and control groups. The number of TUNEL-positive nuclei are shown in red and the arrows indicate the apoptosis nucleus. Scale bars, 50 mm. All data are expressed as the mean ± S.D., n = 3. * *p* < 0.05, ** *p* < 0.01, *** *p* < 0.001. Disclamer: Reprinted with permission from ScienceDirect, Copyright 2024, under a Creative Common License, CC BY-NC-ND 4.0, https://creativecommons.org/licenses/by-nc-nd/4.0/ (accessed on 5 May 2024), Molecular Therapy Nucleic Acids, Vol 16, Xu et al., “Exosomes from Microglia Attenuate Photoreceptor Injury and Neovascularization in an Animal Model of Retinopathy of Prematurity”, https://www.sciencedirect.com/science/article/pii/S2162253119301246 (accessed on 5 May 2024), no changes made [58].

Exosomes are extracellular vesicles, with an average size of 100 nm. They are products of the invagination of cell plasma membranes and may contain various constituents, such as DNA, lipids, and metabolites. These entities with great content and functional heterogeneity are then transported to target cells, and their contents are released. In the context of ROP, XU et al. (2019) hypothesize that microglial-derived exosomes could suppress pro-angiogenic factors and enhance photoreceptor survival. Using electroretinography (ERG), which quantifies electrical activity in the retina following light stimuli, they compared the amplitude of ERG between normal eyes, microglial-derived exosome-treated eyes, and phosphate buffer saline (PBS) controls. Figure 6A illustrates the higher ERG response of healthy eyes and exosome-treated eyes compared to the controls, regardless of the phase (i.e., scotopic and photopic), suggesting improved visual function of the treated cells compared to the controls. The maximal ERG response in the three groups followed a similar trend, with OIR/control eyes having the lowest a-wave and b-wave amplitudes, which are associated with the response of photoreceptors and both muller and bipolar cells, respectively. The underlying mechanism was clarified by a TUNNEL assay, comparing the number of apoptotic nuclei in the retinal outer nuclear layer in the PBS control and exosome-treated groups. They discovered that microglia-derived exosomes suppressed VEGF expression in OIR mice in vivo and reduced the number of apoptotic nuclei in the retinas by over 50% (Figure 6B). The in vitro analysis indicated that the exosomes shuttle miR-24-3p into the photoreceptors, inhibiting hypoxia-induced apoptosis and decreasing angiogenesis through the decrease in angiogenic factors. However, an important obstacle to its advancement in terms of its future application is the difficulty in characterizing and isolating primary human microglial cells [58]. Recently, Li et al. (2023) reported the potential of human umbilical cord mesenchymal stem cell (hucMSC)-derived exosomes in reducing the overexpression of VEGF-A in vitro. HucMSC is an excellent candidate for exosome therapy, due to the simple collection methods involved and its low immunogenicity, amongst others. Though the mechanism underlying the results has yet to be explored, these exosomes downregulated VEGF-A in hypoxic immature human fetal retinal microvascular endothelial cells (hfRMECs). The inhibition of the proliferation of hfRMECs also correlated with the concentration of hucMSC-Exos [59]. Given that the experiments were performed on immature human fetal retinal microvascular endothelial cells acquired from a fetus, these results align specifically with pediatric physiology and pathophysiology. Finally, moving away from ROP therapy, Hu et al. (2022) combined both the exosomes in tears and nanoporous membrane-based resonators as a means to detect biomarkers for ocular diseases efficiently, highlighting encouraging advancements in the realm of molecular diagnostics [60].

### 6.3. Combination Therapy

Emerging treatment modalities involve combined therapy, using laser photocoagulation and anti-VEGF injections for beneficial effects. Autrata et al. (2012) combined diode laser therapy and intravitreal pegaptanib in treating stage 3+ retinopathy in premature infants and demonstrated a lower recurrence rate of neovascularization compared to laser therapy alone. They also deemed this method useful in achieving rapid neovascular regression. However, the systemic and long-term effects have yet to be explored [61]. Similarly, Modrzejewska et al. (2023) compared a combination treatment to ranibizumab monotherapy in infants, for which they found no significant difference in the sequence of administration [62]. As research on anti-VEGF embedded nanoparticles continues to emerge and our understanding progresses, there is potential to implement these in the domain of combination therapy for beneficial results.

Table 1 summarizes the characteristics, outcomes, and stages of nanoparticle use discussed previously.

## 7. Conclusions

Retinopathy of prematurity (ROP) remains a significant cause of visual impairment in premature infants, necessitating ongoing research and innovation in regard to its management. Despite advancements in current treatments, such as cryotherapy, laser photocoagulation, and anti-VEGF therapy, these modalities present notable limitations, including long-term ocular complications, potential ocular tissue damage, and systemic side effects.

Nanotherapeutic approaches offer promising solutions to these challenges. By enabling targeted delivery, enhancing drug permeability, and providing controlled, sustained release of medications, nanotherapies have the potential to improve the safety and efficacy of ROP treatments. These innovations could revolutionize the management of ROP, offering a more effective and safe option for preserving vision and preventing complications in infants suffering from ROP.

As we continue to explore and refine these nanosystems, the future of ROP treatment looks increasingly hopeful. The integration of nanotechnology into ophthalmic care not only underscores the potential for groundbreaking advancements in pediatric eye disease management, but also paves the way for a new era of precision and personalized medicine. The continued development and application of nanotherapeutic approaches holds great promise for enhancing the treatment outcomes and quality of life for children affected by ROP.

## Figures and Tables

**Table 1:** Nanoparticles use in retinopathy of prematurity.

NP	Characteristics	Observations	Stage	Year	Ref.
ROP					
Lipid nanocapsules (LNCs)	Surface cRGD loaded with Cyclosporin A	Prolonged residence time (still detectable 5 days post-injection) Crosses barriers Prevents pathological neovascularization Anti-inflammatory properties Able to target RPE ability Potential pre-emptive treatment	OIR mouse in vivo, intravenous	2022	[34]
Gold NPs	Empty gold NPs	Inhibits angiogenic proliferation, migration, tube formation Inhibits retinal neovascularization in vivo No toxic effects on retinal cells Block VEGF-induced auto-phosphorylation of VEGFR-2 in retinal microvascular endothelial cells	HRMECs in vitro and intravitreal OIR mouse in vivo	2011	[40]
PLGA	VEGF-A165 loaded	Phase 1 treatment Promotes revascularization and prevents severe ROP Ideal clinical administration prior to first ROP exam Normalizes retinal capillary density and arterial tortuosity Reduces venous dilation	Intravitreal OIR mouse	2019	[47]
Folic acid–chitosan-modified mesoporous silica nanoparticles	MiRNA-223 loaded	High stability and loading efficiency Targeted delivery No toxicity in terms of retina or major organs	Microglia/macrophages cells (BV2 and Raw 264.7), HRMECs and HUVECs in vitro, and intravitreal OIR mouse in vivo	2022	[55]
Exosomes	Derived from microglial	Reduce vaso-obliteration and neovascularization Shuttles miR-24-3p to neighboring cells Decreased apoptosis of photoreceptors Difficulty in characterizing and isolating primary human microglial cells	661w cells in vitro and intravitreal OIR mouse in vivo	2019	[58]
Exosomes	hucMSC-Exos	Downregulation of VEGF-A in hypoxic cells Extent of inhibition correlates with concentration	HfRMECs	2023	[59]
Retinal neo-vascularization					
Lipid-like nanoparticles	Loaded with VEGF siRNA smart release	Reduces rate of tube formation, migratory, and proliferative activity of HUVECs Comparable effects to ranibizumab therapy Suppresses VEGF Need for long-term studies	HUVECs in vitro and intravitreal OIR mouse in vivo	2020	[36]
Gold nanodiscs	Optimized anatomy of nanodiscs	Impeccable histology 5 weeks post-injection 14 days to clear nanodiscs Decreased area of neovascular tufts	Intravitreal OIR mouse in vivo	2017	[41]
PLGA	Bevacizumab loaded	No toxic effects reported Increased drug half-life Sustained release of drug More effective in inhibiting endothelial cell proliferation, migration, tube formation Smaller area of neovessel formation	HUVECs in vitro and intravitreal OIR mouse in vivo	2018	[45]
Ischemic retinopathy					
PLGA, PLA	Pazopanib or coumarin-6 loaded	Blocked the angiogenic ability of RMECs, such as cell proliferation and migration	RMECs	2015	[44]
Ocular neo-vascularization					
DOTAP nanoemulsions	Anti-VEGFR loaded oligonucleotides	Successful inhibition of vitreal neovascularization Cationic nanoemulsion promoted drug penetration and endocytosis Cellular inflammation following injection	Intravitreal ROP mouse models in vivo	2012	[54]
PLGA	Loaded with plasmid expressing VLN	VLN-NP inhibits Wnt3a-induced endothelial cell proliferation, migration, tube formation Inhibits abnormal neovascularization in vivo Negative regulator of the WNT pathway Sustained release ≥ 4 weeks	HRMEC in vitro and intravitreal OIR VLDR-/-mouse in vivo	2015	[57]

## Data Availability

Not applicable.
